# Polypharmacy Cut-Off for Gait and Cognitive Impairments

**DOI:** 10.3389/fphar.2016.00296

**Published:** 2016-08-31

**Authors:** Antoine Langeard, Kristell Pothier, Remy Morello, Véronique Lelong-Boulouard, Pascale Lescure, Marie-Laure Bocca, Christian Marcelli, Pablo Descatoire, Chantal Chavoix

**Affiliations:** ^1^Normandie Université, Université de Caen Normandie, Institut National de la Santé et de la Recherche Médicale, COMETECaen, France; ^2^Centre Hospitalier Universitaire de Caen, Departments of Statistics and Clinical Research, Normandie Université, Université de Caen NormandieCaen, France; ^3^Centre Hospitalier Universitaire de Caen, Departments of Pharmacology, Normandie Université, Université de Caen Normandie Université, Institut National de la Santé et de la Recherche Médicale, COMETECaen, France; ^4^Centre Hospitalier Universitaire de Caen, Departments of Geriatrics, Normandie Université, Université de Caen Normandie Université, Institut National de la Santé et de la Recherche Médicale, COMETECaen, France; ^5^Centre Hospitalier Universitaire de Caen, Departments of Rheumatology, Normandie Université, Institut National de la Santé et de la Recherche Médicale, COMETECaen, France

**Keywords:** polypharmacy, cut-off, mobility, gait, cognition, middle age, aging

## Abstract

**Background:** Polypharmacy is a well-established risk factor for falls, and these are one of the major health problems that affect the quality of life as people age. However, the risk of mobility and cognitive impairments consecutive to polypharmacy has been little addressed, despite the association between these adverse outcomes and falls. Moreover, the rare polypharmacy cut-offs were all but one arbitrarily determined.

**Objective:** Studying relationships between polypharmacy and both mobility and cognitive impairments, and statistically determining a cut-off point in the number of medicinal molecule beyond which polypharmacy has deleterious consequences with respect to mobility and cognitive impairment.

**Methods:** We enrolled 113 community-dwelling adults aged 55 years and older with a fall history, with or without injury, in the previous year. We carefully collected information about daily medicinal molecules taken. We assessed basic mobility and global cognition with the Time-Up-and-Go and the Montreal Cognitive Assessment (MoCA) test, respectively (clinicaltrials.gov NCT02292316).

**Results:** Timed-Up and Go test and MoCA scores were both significantly correlated with the number of molecule, used. Receiver Operating Characteristic curves indicate, with high prediction (*p* < 0.002), that daily consumption of five or more molecules is associated with risk for both impaired mobility and global cognition. These relationships were independent of the number of comorbidities and of the pharmacological class.

**Conclusion:** Community-dwelling adults aged 55 years and older who take five or more daily medicinal molecules are at high risk for both mobility and cognitive impairments. Physicians and patients should be aware of these new findings, especially when there are multiple prescribers involved in the care of the patient.

## Introduction

Some drugs are well-known for increasing the risk of falling, especially psychotropics (Hartikainen et al., 2007). Concomitant use of several pharmacological molecules, here called polypharmacy, is also incriminated in falls regardless of their pharmacological class (Tinetti et al., 1988; Ambrose et al., 2013; Fried et al., 2014) because of the deleterious drug–drug interactions that result from interferences between pharmacokinetic and pharmacodynamic properties of the different drugs (Chen et al., 2014). The more drugs people take, the more drug–drug interactions occur (Johnell and Klarin, 2007), and the higher the fall risk is (Kojima et al., 2011). Nevertheless, adverse effects of polypharmacy are seldom taken into consideration by the physicians, partly because of lack of awareness.

Gait and cognitive disorders are two other major risk factors for falls in older adults (Tinetti et al., 1988; Ambrose et al., 2013). However, the number of medicinal molecules that can result in mobility and cognitive impairment has rarely been addressed. A negative linear relationship has previously been reported between the number of medications used and a composite score of balance measures (Agostini et al., 2004). Participants taking five or more medications showed worsened functional abilities over a 7-year follow-up (Pugh et al., 2007). One of the rare studies in the cognitive field showed that incidence of dementia increases steadily with the number of medications used (Lai et al., 2012). The only study that used the Receiver Operating Characteristic (ROC), a powerful tool for risk prediction, to search for a number of medications that could predict cognitive impairment could not find a cut-off value that led to accurate prediction (Gnjidic et al., 2012).

Adverse effects of polypharmacy on dynamic balance and cognition were all observed in populations of over 65 years (Agostini et al., 2004; Pugh et al., 2007; Gnjidic et al., 2012; Lai et al., 2012). Fall risk has, however, been associated with the use of polypharmacy when the study population included younger individuals of 55–65 years (Ziere et al., 2006). This is an important finding since many younger adults are also polymedicated and that occurrence of adverse health outcomes associated with polypharmacy would be even more worrying if occurring so early in life.

The objective of the present study was thus to determine whether, and to what extent, polypharmacy could lead to mobility and cognitive impairments. This was performed in two ways in community-dwelling adults aged 55 years and older: (i) by exploring the relationships between the number of pharmacological molecules taken and both basic mobility and cognitive performance; and (ii) by determining whether there is an optimal number of molecules that could discriminate risk for mobility and cognitive impairment using statistical method for risk prediction.

## Materials and Methods

### Patients

We enrolled 113 individuals over 55 years old who all had experienced one or more falls, either with or without injury, in the year prior to the study. We recruited them among community-dwelling older adults through official bulletin boards at the University hospital in Caen (France) and advertisements in medical offices over a 3-year period. Exclusion criteria assessed by the physicians were: a high-energy fall (e.g., fall down the stairs or from collision), inability to walk for 15 m without help, pathologies that affect posture, drinking more than 21 units of alcohol per week (14 for women), and impaired vision (acuity < 6/10).

The present study was approved by the Lower Normandy Ethics Committee (no. 2011A00556-35; clinical trial registration number: NCT02292316). Each participant provided written informed consent.

### Outcome Measures

We collected information about drugs, vitamins, and supplements taken via medical prescriptions which was confirmed by history taking during the clinical examination. We used the total number of prescribed molecules to estimate medication exposure (e.g., two molecules were counted in case of the combination of two molecules in a single tablet), and the Kaplan–Feinstein scale (Kaplan and Feinstein, 1974) to determine the number of comorbidities.

We assessed basic functional mobility with the Timed-Up and Go test (TUG) that requires standing up from an arm chair, walking 3 m, turning, walking back to the chair, and sitting down. The task was performed twice at a comfortable pace, and we recorded the shortest time to complete the task. We measured global cognition with the Montreal Cognitive Assessment test (MoCA; Nasreddine et al., 2005), a 30-point test that covers six cognitive domains: visuospatial/executive functions, naming, attention, abstraction, recall, and orientation. Cut-off score for impairment was based on the normative reference values corrected for age for the TUG (Bohannon, 2006), and set at 26/30 for the MoCA (Nasreddine et al., 2005). The experimenters who performed the gait and cognitive evaluation were blind to participants’ medical treatment.

### Statistical Analysis

We used adjusted Pearson correlations to analyze relationships between the number of pharmacological molecules taken and mobility score, cognitive score and number of comorbidities. Adjustment variables were age and body mass index (BMI) for the correlations with TUG score, and age only with those with the MoCA score that already takes into account education level.

We performed ROC analyses on TUG and MoCA scores (normal vs. impaired) to determine whether there is an optimal cut-off value for the number of molecules that could be responsible for cognitive and mobility impairments. The area under the ROC curve (AUC) provides a measure of accuracy of the prediction. We then performed several analyses using the optimal cut-off values thus obtained. First, we computed analyses of variance (ANOVAs) to compare TUG score, MoCA score, and the number of psychotropic, antihypertensive, opioid analgesic and others molecules between the participants taking fewer and “as many or more” molecules than the cut-off value. We verified the conditions of validity with Levene’s test. Second, we explored the relationships between the number of molecules prescribed (≥cut-off vs. <cut-off) and (i) TUG score (impaired vs. normal), (ii) MoCA score (<26 vs. ≥26), (iii) the comorbidities (cut-off as obtained in the “Results” Section, and (iv) the interactions between these three variables. We performed both univariate and multivariate logistic regressions on backward selection method. We selected the variables based on the likelihood score statistic, and the adjustment variables were age and BMI when appropriate. We performed all analyses with IBM^®^SPSS^®^22.0 software.

## Results

**Table 1** describes the characteristics of the study population. Mean TUG and MoCA scores were 9.39 ± 3.24 s and 26.47 ± 3.73, respectively, and the number of participants with impaired TUG and MoCA scores was 39 and 27, respectively. The number of pharmacological molecules used was significantly correlated with the TUG score (*R* = 0.322; *p* < 0.001), the MoCA score (*R* = -0.237; *p* < 0.004), and the number of comorbidities (*R* = 0.322; *p* < 0.001). Regarding age of the study population, 34% were younger than 65 years old, and 38% were older than 75 years old.

**Table 1:** Characteristics of the study population (*n* = 113).

Age (mean ±*SD*)	69 ± 8.94
Women	89 (78.7%)
Years of education (mean ±*SD*)	11,19 ± 3.77
BMI (mean ±*SD*)	27,15 ± 5.34
Falls in the previous year (mean ±*SD*)	1.78 ± 1.99
Reason of falls	
Health problem^a^	7 (6.2%)
External events^b^	48 (42.5%)
Lack of attention^c^	50 (44.2%)
Mixed^d^	8 (7.1%)
Falls with no injury	33 (29.2%)
Medication exposure	99 (87.7%)
Molecules taken (mean ±*SD*)	4.42 ± 3.58
Participants by number of molecules taken	
0 molecule	14 (12.4%)
1 or 2 molecules	27 (23.9%)
3 or 4 molecules	22 (19.5%)
5 to 9 molecules	37 (32.7%)
≥10 molecules	13 (11.5%)

*^a^e.g., faintness and fatigue; ^b^e.g., slippery floor, icy conditions, non-visible obstacle in the dark, uneven ground, and inappropriate shoes; ^c^e.g., while performing a secondary task and missing a step; ^d^combination of two or three above reasons.*

**FIGURE 1:**
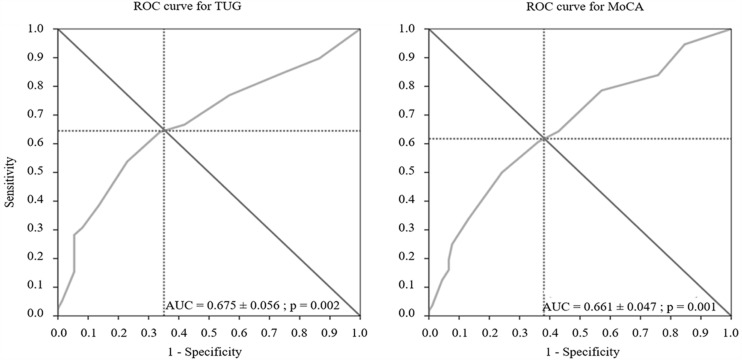
**Each point on the ROC curve corresponds to a specific cut-off, with each cut-off having its own sensitivity and specificity.** The optimal cut-off is defined as the value, here that of the number of molecules, that provides the best combination of sensitivity and specificity. This optimal cut-off can be identified as the intersection of the ROC curve with the diagonal. The area under the curve (AUC) is equal to 1 for perfect discrimination and 0.5 for an uninformative cut-off point. The optimal cut-off value of the number of molecules was 4.5 for each ROC curve. MoCA, Montreal Cognitive Assessment; TUG, Timed Up and Go test.

**Figure 1** that displays the ROC curves shows that the optimal cut-off values was 4.5 for both impaired TUG and MoCA scores with significant AUC for both scores(*p* = 0.002). Participants who took five or more molecules were thus more likely to show impaired TUG and MoCA performance than those who took fewer molecules. When considering this cut-off value, the ANOVAs showed that, compared to the participants taking fewer than five molecules, those taking five molecules and more (1) had significantly lower TUG and MoCA scores (*p* < 0.001 and 0.01, respectively, and (2) took twice as many antihypertensives (0.56 ± 0.11 vs. 1.32 ± 0.12; *p* < 10^-5^), four times more psychotropics (0.20 ± 0.07 vs. 0.56 ± 0.08; *p* < 10^-5^), 10 times more opioid analgesics (0.02 ± 0.04 vs. 0.21 ± 0.04; *p* < 10^-5^), and 2,5 times more molecules classified as others (1.40 ± 0.22 vs. 3.63 ± 0.25; *p* < 10^-5^). Besides, using the ROC methodology with this cut-off of five molecules, we found that participants taking five molecules and more were more likely to have at least two comorbidities (AUC: 0.790 ± 0.044; *p* < 0.001). This cut-off of two comorbidities was further used in the multivariate logistic regression analysis.

Univariate analyses showed that: (i) 64.1% of participants with impaired TUG and 33.8% with normal TUG were taking five or more molecules (*OR* = 3.50, 95% *CI* = 1.55–5.89; *p* = 0.003); and (ii) 59.5% of those with impaired MoCA scores and 35.2% with normal MoCA were taking five or more molecules (*OR* = 2.71, 95% *CI* = 1.23–5.93; *p* = 0.013). Furthermore, multivariate logistic regression analysis (**Table 2**) showed that impaired TUG and MoCA scores, and having two or more comorbidities, were each significantly associated with the use of at least five daily pharmacological molecules; the interactions between TUG score, MoCA score and the number of comorbidities were not significant.

**Table 2:** Results of the logistic regression analysis.

Multivariate logistic regression	Number of molecules ≤5 vs. <5		
	OR	95% CI	*p-*value
MoCA (<26) vs. MoCA (≥26)	3.153	1.187 – 8.373	0.021
TUG (–) vs. TUG (+)	2.753	1.046 – 7.249	0.040
Number of comorbidities (<2 vs. ≥2)	8.730	3.000 – 25.404	<10^-3^

*MoCA, Montreal Cognitive Assessment; TUG, Timed Up and Go.*

## Discussion

To our knowledge, this is the first study to show that impaired mobility and global cognition, assessed here by the TUG and MoCA test, respectively, are both significantly correlated with the number of medicinal molecule taken. ROC curves indicate that daily consumption of five or more molecules results in both impaired TUG and MoCA scores. Logistic regression provides evidence that the relationships between this polypharmacy cut-off and mobility, and between polypharmacy cut-off and cognition, are independent of the relation between mobility and cognition, and of the number of comorbidities.

The poorer TUG score with the increased number of medicinal molecules used confirms the linear relationship between number of medications and impaired balance (Agostini et al., 2004). Furthermore, the reduced basic mobility with concomitant use of five or more medicinal molecules would be consistent with the association of polypharmacy cut-off of five drugs, arbitrary determined, with worsening of gait and balance at 7-year follow-up (Pugh et al., 2007). The present study is the first to define a polypharmacy cut-off value, of five medicinal molecules, for adverse outcomes on functional mobility, as assessed by a static and dynamic balance test, using statistical methods. The significant AUC of the ROC curve indicates that the prediction of impaired mobility when using five or more molecules is very accurate. Finally, the logistic regression analysis supports the validity of this cut-off value since it shows that participants with impaired TUG scores are 3.5 times more likely to be taking five or more pharmacological molecules.

The significant decrease in global cognition with the increased number of medicinal molecules taken is a new finding. It has only been reported that patients with dementia were more likely to use a higher number of medications per day than controls (Lai et al., 2012). Because participants in the present study had essentially normal cognition, our findings extend the deleterious effects of polypharmacy to a mainly cognitively intact population. The prediction of cognitive impairment when again taken five or more molecules was again very good. Excessive polypharmacy (>10 drugs) has been shown to be associated with decline in cognitive capacity as measured by the Mini Mental State Examination (MMSE) in persons over 75 years old (Jyrkkä et al., 2011). However, Gnjidic et al. (2012) did not find evidence of a polypharmacy cut-off that accurately predicted cognitive disorders, as determined by a diagnosis of mild cognitive disorders (MCI) or dementia in participants with impaired MMSE. Discrepancy with the present findings could be explained by the use of the MMSE, known to be less sensitive than the MoCA, for detecting slight cognitive deficits (Nasreddine et al., 2005). Finally, logistic regression analyses showed that participants with impaired MoCA scores are almost three times more likely to have been taking more than five molecules per day than five or fewer. This is thus the first study that, to our knowledge, identifies a polypharmacy cut-off for impaired cognition.

Importantly, logistic regression analyses provide evidence that the relationships between polypharmacy cut-off and mobility, and between polypharmacy cut-off and cognition, are independent of both the relation between mobility and cognition, and the number of comorbidities. Noteworthy, all pharmacological classes are concerned by the present findings and not a specific class only. It should, however, be noted that the participants of the present study who took five or more molecules were the most likely to take drugs known to alter mobility or cognition (antihypertensive, psychotropic, opioid analgesic drugs…). Further investigations are thus required to determine the specific involvement of each class of medicinal molecule in people at risk for cognitive and mobility impairments.

Because our population was younger than those included in most polypharmacy studies [34% between 55 and 64 years vs. almost 100% ≥65 years (e.g., Agostini et al., 2004; Johnell and Klarin, 2007; Pugh et al., 2007; Kojima et al., 2011; Gnjidic et al., 2012; Lai et al., 2012, respectively), our findings highlight that middle aged persons are also affected by the polypharmacy issue. Only rare studies have addressed the issue of the risks of falling (Ziere et al., 2006; Bennett et al., 2014), mobility impairments, in particular during dual walking task (Pothier et al., 2014) or polypharmacy (Bennett et al., 2014; Lu et al., 2015) at different stages of life in older adults. Further investigations would thus be useful for improving our understanding of the deleterious effects of polypharmacy at different stages of adult life in order to implement accurate strategies as soon as polypharmacy becomes a risk to people.

One could argue that the present findings may not be relevant for the general population since all participants had fallen in the previous year. Nevertheless, our population is very similar to the general population in many points: (i) a third did not require a medical consultation since the fall was harmless; (ii) an unavoidable external event was the cause of numerous falls (34.5%); (iii) cognitive and gait abilities were both in the normal range [24% impaired MoCA score vs. 42% in community-dwelling women over 65 years old (Liu-Ambrose et al., 2008); mean TUG score of 9.39 s vs. 9.4 s in the same age group in Bohannon’s meta-analysis (Bohannon, 2006)]; and (iv) the daily number of drugs taken was similar in our sample and in community-dwelling adults aged 60 years and older (4.4 vs. 4.5; e.g., Golden et al., 1999). The only difference from the general population comes from the mode of recruitment since we specifically searched for individuals who had fallen in the previous year. Since one may assume that most people will experience a fall during their life span, after sufficient time has elapsed, one may gather all individuals who would undergo a fall. Because 30–40% of the individuals older than 65 years fall every year (Tinetti et al., 1988), it is thus likely that after our 3 years of inclusion, we had collected a significant part of the general population.

Second, the number of falls and related injury were not considered in the statistical analysis because retrospective self-report in older adults, as recorded in the present study, often results in under-estimation of the true incidence of falls (Cummings et al., 1988), and they were only 29.2% of falls without injury, which limits the interest of intergroup comparisons. The issue of recurrent falls and severity of the falls will be addressed after the 2-years of follow-up presently in progress.

Finally, although our sample size is relatively small, the results of the multivariate logistic regression can be considered as robust because the confidence intervals for the values of the TUG and MoCA are relatively narrow despite this small sample size.

## Conclusion

The present study indicates that the use of five or more pharmacological molecules constitutes a risk for both mobility and cognitive impairments in community-dwelling adults aged 55 years and older, at least in those who experienced a fall. This would likely explain a significant number of falls that occur in older adults, which would be supported by the same polypharmacy cut-off in the number of medications reported for the risk of falling (Gnjidic et al., 2012). This polypharmacy cut-off of five or more molecules should thus be considered by the physicians as a warning signal, especially when there are multiple prescribers involved in the care of the patient. Despite the obvious relation between polypathogy and polypharmacy (Nobili et al., 2011) confirmed here, that makes health status the first determinant of polypharmacy, numerous studies provide evidence for possible reduction in the prescription of medication; for instance, inappropriate medications, insufficient efficacy of highly prescribed drugs and adverse drug–drug interactions (Farrell et al., 2013; Chen et al., 2014). The finding that five medicinal molecules and more are prescribed as soon as a patient shows two pathologies highlights the disproportion between polypathology and number of medicinal molecules taken. Thus, although it may often be impossible to limit the number of molecules used in patients with various and specific pathologies such as after myocardial infarction, physicians and patients should both be aware of these findings.

## Author Contributions

The study was designed by CC and CM. CC was responsible for supervision of the study. PL, CM and PD did the medical visits for inclusions and the collection of clinical data such as medical treatments. They also provided expertise in rheumatology and geriatrics for the results interpretation. M-LB and VL-B’s pharmacological expertise contributed to the literature search and the data interpretation. RM performed the advanced statistical analysis, and thanks to his additional medical expertise, also took an important part in the data interpretation. Gait and cognitive data was collected and analyzed by AL and KP. The paper was written by AL, KP, and CC, and all the authors provided critical revisions of the manuscript for important intellectual content.

## Conflict of Interest Statement

The authors declare that the research was conducted in the absence of any commercial or financial relationships that could be construed as a potential conflict of interest.
